# A cross-country study of mis-implementation in public health practice

**DOI:** 10.1186/s12889-019-6591-x

**Published:** 2019-03-06

**Authors:** Karishma S. Furtado, Elizabeth L. Budd, Rebecca Armstrong, Tahna Pettman, Rodrigo Reis, Pauline Sung-Chan, Zhaoxin Wang, Ross C. Brownson

**Affiliations:** 1 Prevention Research Center, Brown School, Washington University in St. Louis, One Brookings Dr., Campus Box 1196, St. Louis, MO 63130 USA; 2 College of Education, University of Oregon, Eugene, OR 97403 USA; 3 Melbourne School of Population and Global Health, The University of Melbourne, Victoria, 3010 Australia; 4 Hong Kong University of Science & Technology, Clear Water Bay, Kowloon, Hong Kong; 5 Tongji University, Shanghai, China; 6 Department of Surgery (Division of Public Health Sciences) and Alvin J. Siteman Cancer Center, Washington University School of Medicine; Washington University in St. Louis, St. Louis, MO 63130 USA

**Keywords:** Dissemination and implementation, Evidence-based public health, Chronic disease, Mis-implementation, Evidence-based chronic disease prevention

## Abstract

**Background:**

Mis-implementation (i.e., the premature termination or inappropriate continuation of public health programs) contributes to the misallocation of limited public health resources and the sub-optimal response to the growing global burden of chronic disease. This study seeks to describe the occurrence of mis-implementation in four countries of differing sizes, wealth, and experience with evidence-based chronic disease prevention (EBCDP).

**Methods:**

A cross-sectional study of 400 local public health practitioners in Australia, Brazil, China, and the United States was conducted from November 2015 to April 2016. Online survey questions focused on how often mis-termination and mis-continuation occur and the most common reasons programs end and continue.

**Results:**

We found significant differences in knowledge of EBCDP across countries with upwards of 75% of participants from Australia (*n* = 91/121) and the United States (*n* = 83/101) reporting being moderately to extremely knowledgeable compared with roughly 60% (*n* = 47/76) from Brazil and 20% (*n* = 21/102) from China (*p* < 0.05). Far greater proportions of participants from China thought effective programs were never mis-terminated (12.2% (*n* = 12/102) vs. 1% (*n* = 2/121) in Australia, 2.6% (*n* = 2/76) in Brazil, and 1.0% (*n* = 1/101) in the United States; *p* < 0.05) or were unable to estimate how frequently this happened (45.9% (*n* = 47/102) vs. 7.1% (*n* = 7/101) in the United States, 10.5% (*n* = 8/76) in Brazil, and 1.7% (*n* = 2/121) in Australia; *p* < 0.05). The plurality of participants from Australia (58.0%, *n* = 70/121) and the United States (36.8%, *n* = 37/101) reported that programs often mis-continued whereas most participants from Brazil (60.5%, *n* = 46/76) and one third (*n* = 37/102) of participants from China believed this happened only sometimes (*p* < 0.05). The availability of funding and support from political authorities, agency leadership, and the general public were common reasons programs continued and ended across all countries. A program’s effectiveness or evidence-base—or lack thereof—were rarely reasons for program continuation and termination.

**Conclusions:**

Decisions about continuing or ending a program were often seen as a function of program popularity and funding availability as opposed to effectiveness. Policies and practices pertaining to programmatic decision-making should be improved in light of these findings. Future studies are needed to understand and minimize the individual, organizational, and political-level drivers of mis-implementation.

**Electronic supplementary material:**

The online version of this article (10.1186/s12889-019-6591-x) contains supplementary material, which is available to authorized users.

## Background

Chronic diseases like diabetes, cancer, and heart disease are the largest causes of morbidity and mortality worldwide [1, 2]. The field of evidence-based public health, [3–6] namely evidence-based chronic disease prevention (EBCDP) seeks to address the challenge of chronic disease prevention by using the best available scientific evidence, applying program-planning frameworks, engaging the community in decision making, using data and information systems systematically, conducting sound evaluation, and disseminating what is learned [7, 8]. An evidence-based approach to prevention and control can significantly prevent and minimize chronic disease burden [9–11].

However, despite its enhanced ability to address chronic disease, EBCDP is not as widely used as it should be [7, 8, 12]. A considerable amount of the breakdown in the pipeline between evidence production and its application by public health practitioners takes place at the state and local public health levels, which, in the United States and other countries, have substantial authority over protecting the public’s health [13]. Studies have identified barriers impeding evidence-based public health practice at the individual (e.g., lack of EBCDP knowledge), agency/organizational (e.g., absence of leadership support for EBCDP), community (e.g., absence of critical community-based partnerships), sociocultural (e.g., lack of societal demand for evidence-based programs), and political (e.g., lack of buy-in from policymakers) levels in the United States as well as in other developed and developing countries [14–17].

Mis-implementation is defined as the state in which effective interventions are prematurely ended (mis-termination) or, alternatively, ineffective interventions remain in place (mis-continuation). While some literature has examined overuse of clinical interventions in a medical setting, [18–21] few studies have examined mis-implementation in public health [22]. Mis-implementation is likely an important factor in understanding the lag in EBCDP, as it points to the misallocation of resources, and inadequate funding is a commonly-cited barrier to EBCDP [23–25]. Mis-implementation may also be evidence of a culture that does not value or prioritize evidence when making programmatic decisions [26].

This study examined the perceived occurrence of EBCDP program mis-implementation and the most common reasons for program termination and continuation in four countries: Australia, Brazil, China, and the United States. These countries were selected because they represent an array of structures and systems of public health, which make them rich sources of insight into mis-implementation around the world. They also account for a large portion of the world’s chronic disease burden and population [27]. Lastly, the four countries are likely to represent different degrees of experience with EBCDP, based on the greater volume of empirical literature on the topic produced in Australia and the United States relative to Brazil and China [28–37]. We used a quantitative approach in the vein of O’Loughlin et al. [38], who used a survey design to extend the insights of generally case study of multiple case study-based approaches to investigating health promotion program sustainability.

## Methods

*Survey Development* A 22-question, cross-sectional survey was developed based on a literature review of existing measures in EBCDP, [23, 39–41], a guiding framework based on previous work of the research team, [16, 41] as well as information gathered from 50 qualitative interviews of local public health practitioners across the four countries [24, 42]. The resulting instrument contained questions across seven domains derived from previous research on disseminating evidence-based interventions such as awareness of evidence-based public health, adoption of approaches for learning about evidence-based interventions, barriers to and facilitators of implementing evidence-based interventions, and mis-implementation (Additional file 1: Table S1). Where possible (e,g, the domains of awareness of EBCDP interventions and barriers and facilitators of EBCDP implementation), questions were adapted from existing literature. The mis-implementation questions consisted of four of the 22 questions and were novel operationalizations of the mis-termination and mis-continuation constructs, including the frequency of each and reasons for each. New operationalizations were deemed necessary due to the absence of existing options that interrogated the constructs of mis-termination and mis-implementation in a few questions as well as the absence of a gold standard by which to validate concepts of mis-implementation. Instruments examining facets of mis-implementation such as sustainability and de-adoption, which have traditionally been studied in isolation, tend to be longer than was deemed advisable for our instrument, which contained several other domains in addition to mis-implementation [43–47]. For example, the validated Program Sustainability Assessment Tool is 40 items long spread across eight sustainability domains [44]. The response options for the two reason questions were derived from the qualitative interviews as well as literature on common reasons programs are terminated and sustained.

Prior to deployment, 13 chronic disease prevention researchers including one male co-investigator, one female coordinator, and three graduate student research assistants from the United States; two female co-investigators and one female research assistant from Australia; one male co-investigator and one male research assistant from Brazil; and two male and one female co-investigator along with one female research assistant from China reviewed the survey. All of the authors were included among the reviewers. The survey was also forward- and backward-translated to Mandarin and Portuguese from English by members of the research team and pilot tested in each country to ensure contextual appropriateness. As a result, seven response items were found to be inapplicable to participants from China and were excluded from that version of the survey, but included in the versions used in Australia, Brazil, and the United States.

### Study sample

Between November 2015 and April 2016, investigators in each country recruited convenience samples of chronic disease prevention practitioners working primarily at the local and regional levels. Sampling was largely carried out through national databases of chronic disease practitioners, which helped ensure that the geographic diversity of the invited participants reflected the distribution of public health infrastructure in each country. Response rates differed considerably across countries with 18% (*n* = 121/672) of those emailed completing the survey in Australia, 46% (*n* = 76/165) in Brazil, 58% (*n* = 101/174) in the United States, and 87% (*n* = 102/117) in China. Investigators deployed the survey to practitioners through a link embedded in an email. All practitioners provided informed consent. Practitioners in Australia and the United States had the option of accepting a $20 USD gift card for completing the survey. Investigators deemed such financial incentives to be culturally inappropriate in Brazil and China. The ethics review boards of The University of Melbourne, Pontifica Universidade Catolica do Parana, The Hong Kong Polytechnic University, and Washington University in St. Louis approved this study.

### Measures

Participants were first asked a series of sociodemographic and employment history questions (e.g., age category, gender, tenure with their organization, educational credentials). They were then asked to rate their knowledge of EBCDP on a 5-point Likert scale. Two questions operationalized mis-implementation in both its forms (i.e., mis-termination and mis-continuation). These questions asked how often mis-termination and mis-continuation occurred with response options “never,” “sometimes,” “often,” “I do not know,” and “not applicable”. Two more questions then asked for the three most common reasons programs ended and continued with roughly a dozen different response options for each as well as an open-ended “other” option.

### Statistical analysis

To assess bivariate differences in our key outcomes of interest, how often mis-termination and mis-continuation occurred and the reasons for program continuation and termination by country, as well as individual and agency characteristics by country, we used chi-square tests and Fisher’s Exact tests. Fisher’s Exact test was used for contingency tables with expected cell counts of fewer than five. All analyses were conducted using SPSS version 23. Missing data was minimal and excluded from analyses.

## Results

### Sample characteristics by country (Table 1)

The distribution of respondents differed significantly across countries by gender, age, and education (Table 1). Brazil was more evenly split among female and male participants (65.8%, *n* = 50/76) compared with Australia (88.4%, *n* = 107/121), China (71.7%, *n* = 71/102), and the United States (87.1%, *n* = 88/101), whose participants skewed female. Practitioners from Australia, the United States, and Brazil were concentrated and fairly evenly distributed between the ages of 30 and 59. Practitioners from China tended to be younger. Practitioners from Australia and the United States more commonly had advanced graduate degrees. The survey may have been inadequately customized to the educational credentials in Brazil due to the high rate of “other” responses. Most of those who endorsed this option reported working in a public health specialist role. Positions varied widely by country reflecting the diversity of ways in which each country staff public health.

**Table 1:** Differences in Participant and Agency Characteristics by Country

	Australia		Brazil		China		United States		Chi-Sq	*p*-value
	*N* = 121		*N* = 76		*N* = 102		*N* = 101			
Characteristic	%	n	%	n	%	n	%	n		
Participant Demographics										
Female	88.4%	107	65.8%	50	71.7%	71	87.1%	88	32.9	**< 0.0001**
Age									89.0	**< 0.0001**
21–29	20.7%	25	8.2%	6	21.6%	22	6.9%	7		
30–39	33.1%	40	38.4%	28	56.9%	58	21,8%	22		
40–49	14.9%	18	31.5%	23	10.8%	11	28.7%	29		
50–59	20.7%	25	21.1%	16	3.9%	4	28.7%	29		
60+	10.7%	13	3.9%	3	0.0%	0	10.9%	11		
Missing	0.0%	0	0.0%	0	6.7%	7	3.0%	3		
Education/Credentials									92.5	**< 0.0001**
Doctorate	14.2%	17	4.0%	3	0.0%	0	6.9%	7		
Master’s	42.5%	51	32.0%	24	23.5%	24	48.5%	49		
Bachelor’s	30.0%	36	22.7%	17	68.6%	70	27.8%	28		
Other	13.3%	16	41.3%	31	7.8%	8	15.8%	16		
Missing	0.8%	1	1.3%	1	0.0%	0	1.0%	1		
Primary Position										
Physician	37.2%	45	1.3%	1	2.9%	3	41.6%	42	77.4	**< 0.0001**
Community Health Nurse	3.3%	4	13.2%	10	43.1%	44	7.9%	8	73.8	**< 0.0001**
Department Head	5.0%	6	28.9%	22	5.9%	6	14.9%	15	30.2	**< 0.0001**
Nutritionist	0.8%	1	9.2%	7	40.2%	41	0.0%	0	103.5	**< 0.0001**
Statistician	15.7%	19	26.3%	20	3.9%	4	1.0%	1	36.5	**< 0.0001**
Health Educator	13.2%	16	2.6%	2	2.0%	2	18.8%	19	22.5	**< 0.0001**
Division of Bureau Head/Deputy Director	0.0%	0	11.8%	9	0.0%	0	10.9%	11	26.6	**< 0.0001**
Program Manager/Administrator/Coordinator	9.1%	11	1.3%	1	0.0%	0	0.0%	0	22.4	**< 0.0001**
Academic Research	9.1%	11	0.0%	0	0.0%	0	0.0%	0	26.1	**< 0.0001**
Other	1.6%	2	5.2%	4	2.0%	2	2.1%	3	6.9	**< 0.0001**
Missing	5.0%	6	0.0%	0	0.0%	0	2.0%	2	4.2	**< 0.0001**
Agency Features										
Number of Employees									83.8	**< 0.0001**
0–100	37.7%	43	38.0%	27	9.8%	10	56.4%	57		
101–400	20.2%	23	28.2%	20	64.7%	66	24.8%	25		
> 400	42.1%	48	33.8%	24	21.6%	22	17.8%	18		
Missing	5.8%	7	6.7%	5	3.9%	4	1.0%	1		
Size of Population Served									127.0	**< 0.0001**
0–49,999	28.3%	30	29.4%	20	0.0%	0	23.8%	24		
50,000-99,999	10.4%	11	10.3%	7	0.0%	0	24.8%	25		
100,000-399,999	20.8%	22	27.9%	19	81.4%	83	25.8%	26		
> 400,000	40.6%	43	32.4%	22	18.6%	19	23.8%	24		
Missing	12.3%	15	10.5%	8	0.0%	0	2.0%	2		

Boldface indicates significant at alpha < 0.05

### Evidence-based knowledge and Mis-implementation frequency by country (Table 2)

We found significant differences in knowledge of EBCDP across countries with upwards of 75% of participants from Australia (*n* = 91/121) and the United States (*n* = 83/101) reporting being moderately to extremely knowledgeable compared with roughly 60% (*n* = 47/76) from Brazil and 20% (*n* = 21/102) from China (Table 2). Significant differences in perceptions of mis-termination and mis-continuation frequency also existed. Far greater proportions of participants from China thought effective programs were never mis-terminated (12.2% (*n* = 12/102) vs. 1% (*n* = 2/121) in Australia, 2.6% (*n* = 2/76) in Brazil, and 1.0% (*n* = 1/101) in the United States) or were unable to estimate how frequently this happened (45.9% (*n* = 47/102) vs. 7.1% (*n* = 7/101) in the United States, 10.5% (n = 8/76) in Brazil, and 1.7% (*n* = 2/121) in Australia).. The majority of participants from Australia (56.4%, *n* = 68/121) thought mis-termination occurred often, compared to 36.8% (*n* = 28/76) in Brazil and 40.4% (*n* = 41/101) in the United States. Participants from all countries found it more challenging to estimate how frequently programs were mis-continued, with 37.8% (*n* = 46/121) in Australia, 14.5% in Brazil (*n* = 11/76), 52.0% (*n* = 53/102) in China, and 34.5% (*n* = 35/101) in the United States reporting they did not know. The plurality of participants from Australia (58.0%, *n* = 70/121) and the United States (36.8%, *n* = 37/101) reported that programs often mis-continued whereas most participants from Brazil (60.5%, *n* = 46/76) and one third (*n* = 37/102) of participants from China believed this happened only sometimes.

**Table 2:** Differences in Knowledge of EBCDP, Mis-implementation, and Reasons Programs End and Continue by Country

	Australia		Brazil		China		United States			
	*N* = 121		*N* = 76		*N* = 102		*N* = 101		Chi-Sq	*p*-value
Characteristic	%	n	%	n	%	n	%	n		
Knowledgeable of EBCDP									146.7	**< 0.0001**
Not at all	0.8%	1	2.6%	2	15.7%	16	1.0%	1		
Slightly	4.2%	5	2.6%	2	31.4%	32	2.0%	2		
Somewhat	20.2%	24	32.9%	25	32.4%	33	14.9%	15		
Moderately	60.0%	73	44.7%	34	18.6%	19	54.5%	55		
Extremely	15.0%	18	17.1%	13	2.0%	2	27.7%	28		
Mis-implementation										
Frequency of Mis-Termination (Inappropriate Ending)									148.4	**< 0.0001**
Never	1.7%	2	2.6%	2	12.2%	12	1.0%	1		
Sometimes	31.6%	38	39.5%	30	36.7%	37	51.5%	52		
Often	56.4%	68	36.8%	28	5.1%	5	40.4%	41		
Don’t Know	9.4%	11	10.5%	8	45.9%	47	7.1%	7		
Missing	1.7%	2	10.5%	8	1.0%	1	0%	0		
Frequency of Mis-Continuation (Inappropriate Continuation)									241.1	**< 0.0001**
Never	1.7%	2	10.5%	8	11.0%	11	5.9%	6		
Sometimes	0.0%	0	60.5%	46	33.0%	34	19.8%	20		
Often	58.0%	70	7.9%	6	4.0%	4	36.8%	37		
Don’t Know	37.8%	46	14.5%	11	52.0%	53	34.5%	35		
Missing	2.5%	3	6.6%	5	0%	0	3.0%	3		
Reasons Programs End and Continue										
Reasons Programs End (% of times in top 3)^a^										
Grant funding ended	63.6%	77	43.4%	33	24.5%	25	84.2%	85	80.8	**< 0.0001**
Funding diverted to a higher priority program	31.4%	38	31.6%	24	20.6%	21	36.6%	37	6.6	0.085
Change in political leadership	50.4%	61	47.4%	36	8.8%	9	11.9%	12	73.2	**< 0.0001**
Program was evaluated but did not demonstrate impact	22.3%	27	21.1%	16	42.2%	43	9.9%	10	30.0	**< 0.0001**
Opposition/lack of support from policy makers	26.4%	32	28.9%	22	18.6%	19	18.8%	19	4.4	0.219
Program was challenging to maintain	9.9%	12	10.5%	8	48.0%	49	20.8%	21	55.6	**< 0.0001**
Program was never evaluated	19.0%	23	23.7%	18	10.8%	11	15.8%	16	5.6	0.130
Opposition/lack of support from the general public	2.5%	3	21.1%	16	38.2%	39	8.9%	9	56.9	**< 0.0001**
Opposition/lack of support from leaders in my agency	10.7%	13	35.5%	27	13.7%	14	10.9%	11	26.1	**< 0.0001**
A program champion departed	22.3%	27	25.0%	19	5.9%	6	9.9%	10	19.1	**< 0.0001**
Program was expensive	5.8%	7	11.8%	9	15.7%	16	8.9%	9	6.3	0.098
Program was not evidence-based	3.3%	4	23.7%	18	12.7%	13	3.0%	3	29.4	**< 0.0001**
Program was adopted or continued by other organizations	4.1%	5	2.6%	2	2.0%	2	13.9%	14	16.9	**0.001**
Insurance funding/coverage ended	1.7%	2	9.2%	7	0.0%	0	7.9%	8	14.5	**0.002**
Reasons Programs Continue (% of times in top 3)^a^										
Sustained support from policymakers	27.3%	33	43.4%	33	31.4%	32	22.1%	22	11.6	**0.009**
Sustained funding	28.1%	34	39.5%	30	36.3%	37	35.6%	36	3.2	0.358
Sustained support from leaders in your agency	27.3%	33	18.4%	14	35.3%	36	24.8%	25	6.7	0.084
Absence of alternative options	28.1%	34	26.3%	20	22.5%	23	17.8%	18	3.6	0.310
Program was never evaluated	33.1%	40	35.5%	27	8.8%	9	16.8%	17	27.1	**< 0.0001**
Sustained support from the general public	15.7%	19	21.1%	16	37.3%	38	15.8%	16	18.7	**< 0.0001**
Program was easy to maintain	24.0%	29	18.4%	14	21.6%	22	23.8%	24	1.0	0.799
Presence of a program champion	23.1%	28	28.9%	22	13.7%	14	21.8%	22	6.3	0.096
Program was low-cost	19.0%	23	18.4%	14	8.8%	9	18.8%	19	5.6	0.135
Prohibitive costs of starting something new	13.2%	16	9.2%	7	9.8%	10	6.9%	7	2.5	0.473
Program was considered evidence-based	10.7%	13	3.9%	3	16.7%	17	6.9%	7	9.3	**0.026**

Boldface indicates significant at alpha < 0.05

^a^The original series of questions asked participants to select the three most frequent reasons from the lists above

### Reasons programs end and continue by country

To provide context to our examination of mis-implementation, we asked participants to select from a list (or suggest an alternative) the three most common reasons why programs ended and continued (Table 2). We documented a handful of nearly “universal” (i.e., commonly-cited across all countries) reasons for program termination including funding ending or being diverted and a lack of support from key stakeholders. In addition to these reasons, practitioners from Australia and Brazil reported that changes in political leadership often led to program termination (50.4%, *n* = 61/121 and 47.4%, *n* = 36/76 respectively). Among participants from Brazil, lack of support from agency leadership was also one of the most frequently cited reasons for programs ending (35.5%, *n* = 27/76). China’s top reasons differed significantly from the other countries’ and included that programs were difficult to maintain (48.0%, *n* = 49/102), programs were not demonstrating impact (42.2%, *n* = 43/102), and lack of support from the public (38.2%, *n* = 39/102). In the United States the prevailing issue was by far funding ending (84.2%, *n* = 85/101) or being diverted (36.6%, *n* = 37/101).

We observed less within-country consensus on why programs continued, as indicated by the fact that no single reason was endorsed by the majority of participants in any country. However, some of the same reasons did rise to the top across countries including sustained funding, the absence of alternative options, sustained support from agency leadership, and programs that were easy to maintain. Sustained support from policymakers seemed to be particularly influential for keeping programs running in Brazil, with 43.4% (*n* = 33/76) of participants citing this reason. Sustained support from the general public was a top reason for continuing programs in China (37.3%, *n* = 38/102) but not in Australia (15.7%, *n* = 19/121), Brazil (21.1%, *n* = 16/76), or the United States (15.8%, *n* = 16/101).

## Discussion

Mis-implementation is an under-studied barrier to evidence-based practice. While de-adoption is being studied in the clinical space, where it goes by some four dozen names, [20, 21] less attention has been paid to it in the public health arena. In the field of public health, sustainability, or the continuation or discontinuation of a program or intervention once implemented and after the initial funding has ended, [48, 49] aligns to one half of mis-implementation. The dual nature of mis-implementation seems to be unexplored even in the domain of evidence-based medicine, where the focus is on disinvestment in low-value clinical practices [18–21].

We assert that mis-implementation is a two-sided practice that refers both to the de-adoption of effective programs, policies, or interventions (i.e., “mis-termination”) and to the continuation of ineffective programs, policies, or interventions that should end (i.e., “mis-continuation”). This exploratory study is likely the first to examine mis-implementation in both of its forms in an applied public health setting in multiple countries.

Our results suggest that mis-implementation occurs quite often and that mis-termination is more common—or more visible—than mis-continuation. Over 70% of practitioners surveyed in Australia, Brazil, and the United States reported that mis-termination happened sometimes or often. Among American practitioners, 40% (*n* = 40/101) thought mis-termination occurred often and 36.8% (*n* = 37/100) thought mis-continuation happened often. These findings generally support the only other published study to the authors’ knowledge that has examined mis-implementation in public health [22]. This cross-sectional study of over 900 public health practitioners at state and local public health departments found similar rates of mis-termination and mis-continuation with reasons for each differing somewhat at the state versus local level.

Interestingly, mis-continuation seemed to happen less often across all countries, with 37–68% of participants (*n* = 70/121 in Australia, *n* = 52/76 in Brazil, *n* = 34/102 in China, and *n* = 57/101 in the United States) reporting that it happened often or sometimes. This could point to a particular struggle with sustainment in the delivery of public health at the local level [50, 51]. However, the difference could also reflect a greater difficulty identifying mis-continuation relative to mis-termination. Indeed, a greater portion of practitioners across all countries did not know how often mis-continuation occurred compared to mis-termination. Mis-termination involves recalling instances when things came to an end, which is likely inherently more memorable than that the absence of such an ending (i.e., mis-continuation). This potential recall bias should be considered as research in the area of mis-implementation progresses and measures are optimized.

Practitioners from China were both more optimistic and more uncertain about the occurrence of mis-implementation relative to their colleagues in other countries. A greater proportion of them than in any other country thought mis-termination and mis-continuation never happened. However, the plurality of Chinese participants were unable to gauge how often either type of mis-implementation occurred. The top-down culture in China’s public health system may make observing mis-implementation more difficult. The participants from China predominantly worked for government-run hospitals. Because of the centralized health planning model used in China, wherein the central government has overall responsibility for national health policy and administration, local practitioners may be less involved in determining whether and why programs continue or end. Officials working in such an environment might not know how often mis-implementation occurs or might assume that programs are continuing or ending for good reasons (i.e., that mis-implementation does not occur often).

It is also worth noting that practitioners from China self-reported significantly lower knowledge of EBCDP, and that lack of knowledge might impede their ability to identify mis-continuation and mis-termination. The lower ratings may also reflect cultural differences in willingness to claim expertise in something. In Australia and the United States, where the large majority of participants tended to rate their knowledge to be moderate or excellent, mis-implementation was perceived as occurring far more often. This aligns with literature reporting that a country’s development status can predict structural differences in the provision of public health measures and clinical healthcare that influence their program implementation outcomes and their awareness of evidence-based practices [52–54]. Further research should investigate whether the positive correlation between knowledge and perceived rate of mis-implementation persists at the individual level and when controlling for other factors.

Consideration of the reasons participants gave for programs continuing and ending brings the phenomenon of mis-implementation into greater focus*.* “Grant funding ending” was the most commonly-cited reason for programs ending in Australia and the United States and the second most-common reason in Brazil. This reflects the growing concern around sustainment, or the continuation of a program once implemented and generally after initial funding from federal or state agencies has been exhausted [17]. In addition to funding, changes in political leadership and changes in priorities (which are often dictated by political authorities) were also common reasons programs end that align with the literature base [19, 22]. Reviews of the phenomenon of sustainment similarly find that organizational capacity, in addition to context, processes, and other factors influence whether a program is maintained [48, 55]. Scheirer [49] discusses three categories of factors that affect sustainability beyond securing new funding including aspects of project design and characteristics (e.g., whether the program is modifiable to meet local need), factors within the organizational setting (e.g., the presence of a program champion), and factors in the broader community environment (e.g., support from external community leaders. As found by Scheirer and confirmed by this study, staff tend to focus on challenges securing replacement funding as the primary obstacle to sustainment, potentially at the exclusion of some of these other factors.

Just as interesting as the most commonly cited reasons for program termination are the *least* commonly cited reasons. In both Australia and the United States, not being evidence-based was rarely the reason a program ended, which underscores the phenomenon of mis-continuation. Similarly, in Brazil and China, programs infrequently ended because they were picked up by other organizations, a viable approach to sustainment. Perhaps the most legitimate reason for a program to end is because it was evaluated and did not demonstrate impact. Less than a quarter of practitioners in Australia, Brazil, and the United States cited this as a top-three reason, suggesting that programs that end due to lack of funding, or lack of support, or any of the other most common reasons, are often terminated without a clear sense of whether they are effective.

Practitioners from all countries agreed that having sustained support from various key stakeholders (e.g., policymakers, agency leadership) was among the top reasons programs continued. Several practitioners from Australia and the United States used the open-ended response option to point to practitioner preferences and attachment to programs leading to the continuation of those programs. Sustained funding, the absence of alternatives, and ease of maintenance also led to the continuation of programs. Again, not being evidence-based or evaluated for effectiveness were amongst the least common reasons programs ended across all four countries.

While there was consistency in reasons programs end, the cross-country differences point to important contextual differences in the culture and structure surrounding public health that are important to keep in mind and further explore when seeking to enhance evidence-based public health around the world. In Brazil, for example, policymakers seem to be particularly influential at determining whether programs end and continue. There, a shift in political leadership was the top reason programs end and sustained support from policymakers was the most common reason programs continued. The support of agency leadership and program champions was also key. Practitioners from China reported that the support of the public was critical to keeping programs in place. In both Brazil and China, EBCDP seems to be in a more nascent stage than in Australia and the United States, as reflected by the greater degrees to which Brazil and China rely upon support from various stakeholder groups compared to the more autonomous systems in Australia and the United States and lower levels of self-attested knowledge of EBCDP. These differences in influences will be important to acknowledge when crafting strategies to improve evidence-based implementation in different countries.

Despite the cross-country differences, however, the prevailing theme from this study is that, across all countries, decisions about ending and continuing programs often seem to be made with incomplete consideration of whether the program in question was evidence-based or demonstrating impact. Instead decisions seem to be made based on what can be funded, what has support from key stakeholders, and how easy it is to maintain the status quo relative to the challenge of starting something new. These findings have potential implications for public health policy and practice. Decisions regarding the continuation or termination of programs should be at least partly a function of their impact and evidence base in addition to other more political and logistical/efficiency factors. These decisions should also be made in a transparent manner to ensure that staff have visibility into how program commitments are made or withdrawn. Such transparency may encourage greater adherence and to decision-making protocols and accountability.

### Limitations

The findings reported here are exploratory and should be considered in light of the study’s limitations. We relied on a small set of questions pertaining to perceptions of mis-implementation, program termination and continuation, and knowledge of EBCDP that have not yet been psychometrically tested or independently validated against a gold standard. Selection bias is quite possible, given the non-randomized nature of the study, the adaptations to sampling strategies to accommodate country-specific differences, and the widely ranging response rates. While the survey instrument was forward- and backward-translated from English to Mandarin and Portuguese to ensure fidelity, some concepts and responses may have been lost in translation given the substantial social, cultural, and structural differences between the four countries. Self-reported perceptions of the frequency of and reasons for mis-implementation are also susceptible to recall bias. Additionally, perceptions of mis-implementation may vary by a number of individual and organizational factors, including tenure in position, job responsibilities, programmatic area, and organizational structure, some of which this study examined, but none of which were included in a multivariable model predicting mis-implementation due to small cell sizes.

## Conclusions

Mis-implementation by definition involves the mis-allocation of scarce public health resources. This is the first cross-national study with standardized methods to examine patterns in mis-implementation. It found that public health practitioners across four diverse countries perceive mis-implementation fairly regularly as they seek to prevent chronic diseases at the local levels. While the reasons programs end and continue inappropriately vary from country to country, they generally support the common theme that the culture of public health practice seems to be too often focused on what is easy, familiar, and appealing to external stakeholders as opposed to what is impactful, evidence-based, or challenging. Future studies are needed to examine in closer detail the individual, organizational, and political-level predictors of mis-implementation as well as approaches to minimizing this mis-use of limited resources.

## Additional file

Additional file 1: **Table S1.** Survey Instrument. (DOCX 21 kb)

## Abbreviations

EBCDP: Evidence-based chronic disease prevention
