# Supplementary material for: A cross-country study of mis-implementation in public health practice
Source: BMC Public Health. 2019 Mar 6;19:270. doi: 10.1186/s12889-019-6591-x (PMC6404329; doi:10.1186/s12889-019-6591-x)
Supplement: Supplementary file 1 — Table S1. Survey Instrument. (DOCX 21 kb) [file 12889_2019_6591_MOESM1_ESM.docx]

| **Supplementary Table: Survey Instrument** | |
| --- | --- |
| **Questions** | **Response Options** |
| **Awareness** |  |
| - Evidence-based public health is defined as: “the process of integrating science-based interventions with community preferences to improve the health of populations” (Kohatsu et. al, 2004).  1. With this definition in mind, how knowledgeable are you with evidence-based processes? (*select one*) | - Not at all knowledgeable - Slightly knowledgeable - Somewhat knowledgeable - Moderately knowledgeable - Extremely knowledgeable |
| **Adoption** |  |
| Definition: Evidence-based interventions are those that several studies have found to be effective at preventing chronic disease. Repositories are collections of evidence-based interventions (e.g., Guide to Community Preventive Services) (US), Health-Evidence.org (Australia), Cochrane Collaboration (US, Australia)).   1. I have used repositories to find evidence-based interventions: (*select one*) | - in none of my programmatic areas - in a few of my programmatic areas - in many of my programmatic areas - in all of my programmatic areas |
| 1. Staff at my agency use repositories of evidence-based interventions:   (*select one*) | - in none of my programmatic areas - in a few of my programmatic areas - in many of my programmatic areas - in all of my programmatic areas |
| 1. When you make decisions about such things as program planning and implementation, policy development, or funding, which of the following are important to you? (*select the top three*) | - Support from leadership at my agency - Support from elected officials - Support from community partnerships - Recommendations from the funding agency - Colleagues are using the intervention - Available resources (program dollars & staff) - How easy the intervention or policy is to implement - Evidence regarding the effectiveness of the intervention - Health planning tools (e.g. MAPP or Health People 2010) - Relevance of the intervention to the population of interest - Seriousness of the health problem - Other, please specify ______ - Not applicable |
| 1. What avenues do you use to learn about the current study findings on evidence-based chronic disease prevention interventions? (*select all that apply*) | - Academic journals - Conferences - Email alerts - Evidence-based repositories - Facebook - Funders ^a^ - Government agency staff - Government reports - Internet search engines - Listservs/Newsletters/Online forums - Media campaigns/Media interviews - Networks - Partnerships (e.g., with universities, health departments, professional associations) - Policy briefs ^a^ - Press releases - Stakeholders ^a^ - Technical assistance/Data liaison - Trainings/Workshops/Meetings within my agency - Webinars - Other, please specify ______ - None |
| 1. For which avenues would you like additional access? (*select all that apply*) | - *Same responses as #13* |
| **Implementation** |  |
| 1. Approximately what percentage of programs supported by your agency would you say are evidence-based? | *Fill in the blank 0-100%* |
| 1. As you think about the future, what is one thing you would change to help you implement evidence-based chronic disease prevention interventions? | - *Fill in the blank* |
| **Maintenance** |  |
| Quality improvement (QI) refers to ongoing formal assessments of the effectiveness and quality of public health chronic disease prevention efforts. (NACCHO, 2015).  Some examples of quality improvement processes include: Results-based accountability (RBA), Community Health Improvement Plan (CHIP), Plan-Do-Study-Act (PDSA), and Plan-Do-Check-Act.   1. Staff at my agency use quality improvement processes: (*select one*) | - in none of my programmatic areas - in a few of my programmatic areas - in many of my programmatic areas - in all of my programmatic areas |
| 1. In your opinion, how often do programs end that should have continued? (i.e., end without warrant) (*select one*) | - Never - Sometimes - Often |
| 1. When you think about public health programs that have ended, what are the most common reasons for programs ending? (*Select the top three*) | - Program was never evaluated - Program was evaluated but did not demonstrate impact - Opposition/lack of support from leaders in my agency - Opposition/lack of support from the general public - Opposition/lack of support from policy makers - Funding diverted to a higher priority program - Grant funding ended - Change in political leadership - Insurance funding/coverage ended - Program was adopted or continued by other organizations - A program champion departed - Program was not evidence-based - Program was expensive - Program was challenging to maintain - Other, please specify ______ - I do not know - Not applicable |
| 1. In your opinion, how often do programs continue that should have ended? (i.e., continue without warrant) (*select one*) | - Never - Sometimes - Often |
| 1. When you think about public health programs that continued that should have ended, what are the most common reasons for their continuation? (i.e., continue without warrant) (*Select the top three*) | - Program was never evaluated - Sustained support from leaders in your agency - Sustained support from the general public - Sustained support from policymakers - Prohibitive costs of starting something new - Absence of alternative options - Sustained funding - Presence of a program champion - Program was considered evidence-based - Program was low-cost - Program was easy to maintain - Other, please specify ______ - I do not know - Not applicable |
| - **Contextual Factors** |  |
| 1. Which of the following are personal barriers that make it harder for you to select and implement evidence-based chronic disease prevention interventions? *(Select all that apply*) | - Not being an expert on relevant issues - Lack of confidence in finding data and statistics - Lack of skills to develop evidence-based interventions - Lack of confidence in carrying out evidence-based interventions - Lack of decision-making authority - Low value of evidence-based approaches - Workload is too heavy/not enough time - Overwhelmed by task - Other, please specify ______ - None |
| 1. Which of the following are agency-level barriers that make it harder for you to select and implement evidence-based chronic disease prevention interventions? (*Select all that apply*) | - Poor understanding of evidence-based approaches - Culture/climate is not supportive of change/new ideas - No existing policies to support evidence-based approaches - Agency does not provide training in evidence-based approaches - Staff/leaders lack formal training in evidence-based approaches - Lack of access to resources (e.g., computer, Internet) - Not enough funding - Low priority placed on chronic disease prevention - No systems to ensure interventions are evidence-based - Not enough staff - Beliefs that evidence-based interventions are too difficult to implement/sustain - Other, please specify ______ - None |
| 1. Which of the following are community-level barriers that make it harder for you to select and implement evidence-based chronic disease prevention interventions? (*Select all that apply*) | - Lack of access to repositories/databases of scientific studies - Lack of partnership between agency and community - Community members’ needs compete with evidence-based recommendations - Catering to preferences of funders ^a^ - Low priority placed on chronic disease prevention - Other, please specify ______ - None |
| 1. Which of the following are sociocultural barriers that make it harder for you to select and implement evidence-based chronic disease prevention interventions? (*Select all that apply*) | - Distrust of scientific data in the populations served - Community cultural practices conflict with evidence-based recommendations - Not enough relevant evidence for populations served - Serving a rural setting where data are lacking ^a^ - Serving a highly disadvantaged population - Serving a population that speaks a language different from the majority ^a^ - Evidence is presented in a language I do not understand - Other, please specify ______ - None |
| 1. Which of the following are political/economic barriers that make it harder for you to select and implement evidence-based chronic disease prevention interventions? (*Select all that apply*) | - Political leaders not providing enough support - Funding changes that occur with changes in political leadership - Political climate conflicts with evidence-based chronic disease prevention recommendations - Health care system does not support evidence-based chronic disease prevention - Other, please specify ______ - None |
| 1. For which of the following skills would you like additional technical support or training? (*Check all that apply*) | - Prioritizing program and policy options - Quantifying the public health issue using descriptive epidemiology (e.g. concepts of person, place, time) - Using quantitative evaluation approaches (e.g. surveillance or surveys) - Using qualitative evaluation approaches (e.g. focus groups, key informant interviews) - Developing an action plan for achieving goals - Defining the health issue according to the community’s needs and assets - Adapting interventions for different communities and settings - Using economic data in the decision making process - Communicating research to policy makers - Other, please specify ________ - None |
| **Individual and agency characteristics** |  |
| 1. What is your gender? (*select one*) | - Male - Female - Other - Prefer not to answer |
| 1. What is your age? (*select one*) | - 21-29 - 30-39 - 40-49 - 50-59 - 60 and over - Prefer not to answer |
| 1. What degree/credentials do you hold? (*Check all that apply*) | - BS/BA - CHES - Certified Health Educator (in Diabetes, Asthma, etc.) - RN or RD - MS or MSc - MPH or MSPH - MA - Other Master’s degree - NP - MO or DO - PhD, DrPH, ScD - Other, please specify ______ |
| 1. Though you may work in several capacities, how do you best describe your primary position? (*select one*) | - Academic Researcher - Academic Educator - Community Health Nurse - Department Head - Division or Bureau Head/ Division Deputy - Director - Epidemiologist - Health Educator - Nutritionist/ Dietician - Physician - Program Manager/Administrator/Coordinator - Program Planner/ Evaluator - Public Health Specialist - Social Worker - Statistician - Other, please specify ______ |
| 1. The agency in which I work has the following number of employees. (*select one*) | - 0-50 - 51-100 - 101-200 - 201-400 - 401-800 - >800 - I do not know |
| 1. The size of the population my agency serves is has the following number of people. (*select one*) | - 0-24,999 - 25,000-49,999 - 50,000-74,999 - 75,000-99,999 - 100,000-149,999 - 150,000-199,999 - 200,000-299,999 - 300,000-399,999 - 400,000+ - I do not know |
| 1. Is there anything else you would like to share on the topic of evidence-based chronic disease prevention? Please specify. | *Fill in the blank* |
